# New Geographical Insights of the Latest Expansion of *Fusarium oxysporum* f.sp. *cubense* Tropical Race 4 Into the Greater Mekong Subregion

**DOI:** 10.3389/fpls.2018.00457

**Published:** 2018-04-09

**Authors:** Si-Jun Zheng, Fernando A. García-Bastidas, Xundong Li, Li Zeng, Tingting Bai, Shengtao Xu, Kesuo Yin, Hongxiang Li, Gang Fu, Yanchun Yu, Liu Yang, Huy Chung Nguyen, Bounneuang Douangboupha, Aye Aye Khaing, Andre Drenth, Michael F. Seidl, Harold J. G. Meijer, Gert H. J. Kema

**Affiliations:** ^1^Agricultural Environment and Resources Institute, Yunnan Academy of Agricultural Sciences, Kunming, China; ^2^Bioversity International, Kunming, China; ^3^Wageningen University and Research, Wageningen Plant Research, Wageningen, Netherlands; ^4^Wageningen University and Research, Laboratory of Phytopathology, Wageningen, Netherlands; ^5^Xishuangbanna Dai Autonomous Prefecture, Plant Quarantine and Protection Station, Jinghong, China; ^6^Institute of Microbiology, Guangxi Academy of Agricultural Sciences, Nanning, China; ^7^Institute of Tropical and Subtropical Industry Crops, Yunnan Academy of Agricultural Sciences, Kunming, China; ^8^Institute of Biotechnology, Guangxi Academy of Agricultural Sciences, Nanning, China; ^9^Plant Protection Research Institute, Vietnam Academy of Agricultural Sciences, Hanoi, Vietnam; ^10^Ministry of Agriculture & Forestry, National Agriculture & Forestry Research Institute, Horticulture Research Center, Vientiane, Laos; ^11^Biotechnology Research Department, Mandalay, Myanmar; ^12^Centre for Horticultural Science, The University of Queensland, Brisbane, QLD, Australia

**Keywords:** Laos, Myanmar, Vietnam, China, Fusarium wilt, single nucleotide polymorphism (SNP), phytogeography, The Greater Mekong Subregion (GMS)

## Abstract

Banana is the most popular and most exported fruit and also a major food crop for millions of people around the world. Despite its importance and the presence of serious disease threats, research into this crop is limited. One of those is Panama disease or Fusarium wilt. In the previous century Fusarium wilt wiped out the “Gros Michel” based banana industry in Central America. The epidemic was eventually quenched by planting “Cavendish” bananas. However, 50 years ago the disease recurred, but now on “Cavendish” bananas. Since then the disease has spread across South-East Asia, to the Middle-East and the Indian subcontinent and leaped into Africa. Here, we report the presence of *Fusarium oxysporum* f.sp. *cubense* Tropical Race 4 (Foc TR4) in “Cavendish” plantations in Laos, Myanmar, and Vietnam. A combination of classical morphology, DNA sequencing, and phenotyping assays revealed a very close relationship between the Foc TR4 strains in the entire Greater Mekong Subregion (GMS), which is increasingly prone to intensive banana production. Analyses of single-nucleotide polymorphisms enabled us to initiate a phylogeography of Foc TR4 across three geographical areas—GMS, Indian subcontinent, and the Middle East revealing three distinct Foc TR4 sub-lineages. Collectively, our data place these new incursions in a broader agroecological context and underscore the need for awareness campaigns and the implementation of validated quarantine measures to prevent further international dissemination of Foc TR4.

## Introduction

Panama disease or Fusarium wilt is caused by the soil-borne fungus *Fusarium oxysporum* f.sp. *cubense* (Foc), and was first described in Australia in 1874 (Bancroft, 1876). The fungus penetrates the roots and from there colonizes the vascular system of the banana plant. Together with the plant responses, this results in occlusion of the xylem vessels which causes wilting and eventually death of infected plants (Guo et al., 2014). The decimation of susceptible “Gros Michel” bananas that were grown in large-scale monoculture plantations in Central America during the 1900s earned Fusarium wilt its reputation as a pathogen of global significance. Losses of “Gros Michel” were first recognized in Central America (Costa Rica and Panama) in 1890, and were soon reported in Africa, the Caribbean, and South America (Ploetz, 2015). The Fusarium wilt epidemic was caused by a set of Foc strains that are collectively called Race 1 and decimated the large-scale monocultures of “Gros Michel” on which the banana industry in America relied. No effective control methods were found other than replacing “Gros Michel” with resistant “Cavendish” bananas in Central America during the 1960s. This replacement has been highly successful to quench the Fusarium wilt epidemic that was caused by Foc Race 1 strains. Since then, “Cavendish” production expanded into large global monocultures, which are evidently prone to disease threats, including black Sigatoka and Panama disease (Ordoñez et al., 2015b; Arango Isaza et al., 2016; Diaz-Trujillo et al., 2018). However, this has not resulted in global research efforts to neutralize these problems. Therefore, another genetic lineage of Foc [vegetative compatibility group (VCG) 01213], colloquially called Tropical Race 4 (Foc TR4), which originates from Indonesia and affects many banana varieties, including those belonging to the “Cavendish” group, has now developed into a global threat (Ordoñez et al., 2015b). It has spread to five Asian “Cavendish”-producing countries and Australia, and was recently also discovered in the Middle East, the Indian subcontinent and Africa (García-Bastidas et al., 2014; Ordonez et al., 2015a; Ploetz et al., 2015; Promusa, 2016). Therefore, the global banana industry is under serious threat by this soil-borne fungal disease (Ploetz and Churchill, 2011; Pocasangre et al., 2011; Shabani et al., 2014; Ordoñez et al., 2015b), and its recent rapid spread has raised international concerns with regard to future food security in the tropics and sustainability of the international banana trade that is nearly exclusively based on “Cavendish” clones (D'hont et al., 2012; FAO, 2014). Currently, “Cavendish” clones comprise 15% of the global banana production but they are increasingly gaining importance for domestic markets. Presently, they occupy ~40% (Ploetz et al., 2015) of the total global area. Clearly, this comes with a huge risk for a pandemic as these clones are susceptible to Foc TR4. The vegetative propagation of planting material and a lack of diversification efforts over the last century have increased the genetic vulnerability of the crop to unacceptable levels, which threatens food security. This urges for international, regional, and local measures aimed at prevention and management of this destructive disease (Ploetz, 2015).

The biological complexity of soil-borne diseases—with surviving propagules that remain viable for decades—and taking into account the historical track-record of Foc (Li et al., 2013), demonstrates that disease management has proven to be difficult (Ploetz, 2015). Hence, prevention is currently the major strategy to avoid new Foc TR4 incursions. In 1967, Foc TR4 surfaced in Taiwan, supposedly after introduction of infected plants from Sumatera, Indonesia (Su et al., 1977; Hwang and Ko, 2004). From there, it has disseminated likely into the Chinese province of Fujian, and then gradually to Guangdong, Guangxi, Hainan and finally in 2009 to Yunnan (Sun et al., 1978; Su et al., 1986; Hwang and Ko, 2004; Qi et al., 2008; Buddenhagen, 2009; Li et al., 2013). The expansion of Foc TR4 was facilitated by new large scale “Cavendish” production practices across this area along with limited awareness and lacking quarantine measures. The cultivation of “Cavendish” now shifts to Laos, Myanmar, Vietnam, and other countries in the Greater Mekong Subregion because of limited suitable land for banana production to meet the increasing market demand. During a survey in Vietnam, Laos, and Myanmar in March and May 2016 we observed the presence of Fusarium wilt in “Cavendish” plantations. Here, we provide details on the regional and international expansion of Foc TR4, which is worrisome as it threatens both food security and the international trade (Ordonez et al., 2015a; Ploetz et al., 2015; Mostert et al., 2017).

## Materials and methods

### Sample collection

**Figure 1:**
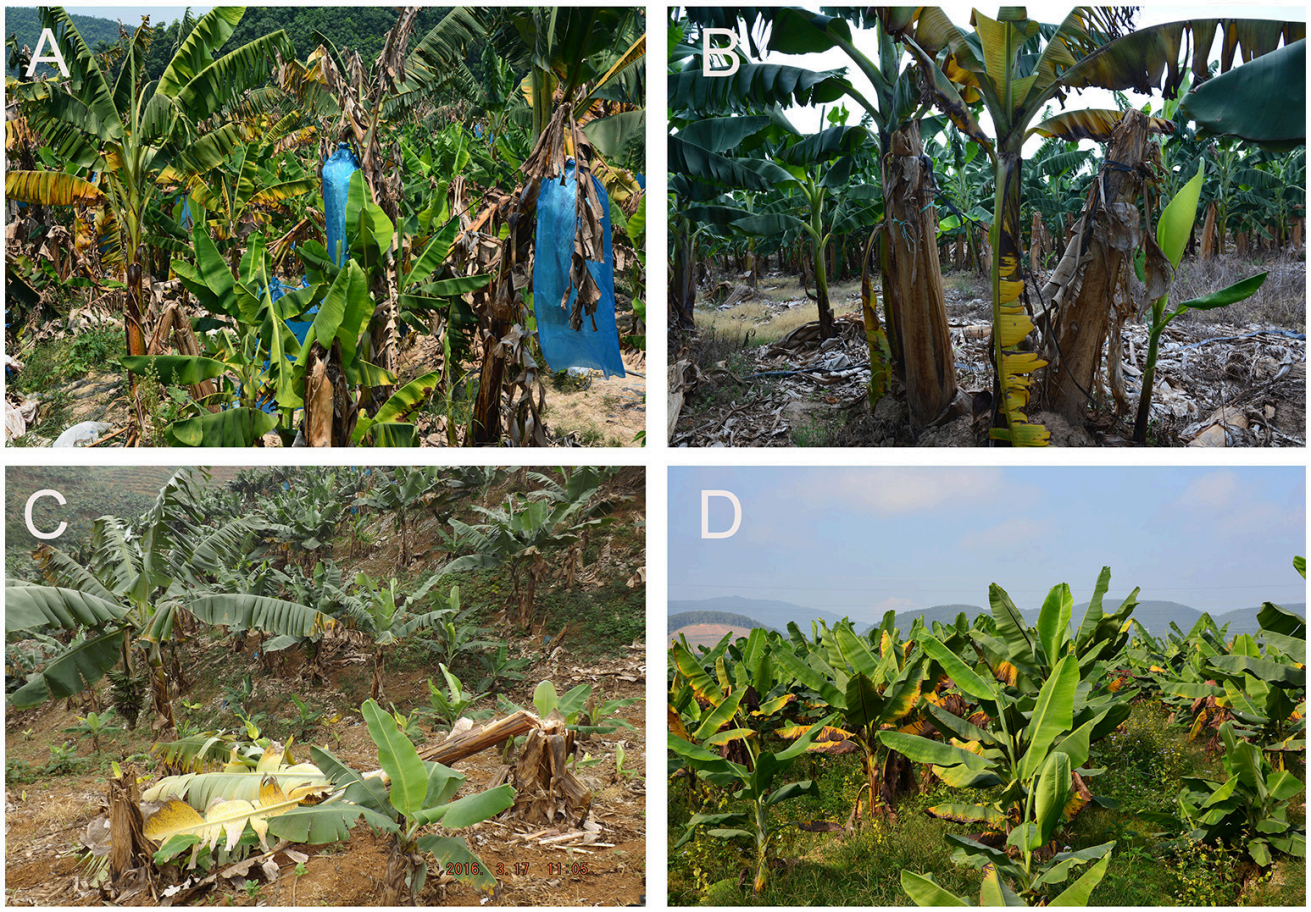
Banana plants with Fusarium wilt symptoms in sampled “Cavendish” plantations in Laos **(A)**, Myanmar **(B)**, Vietnam **(C)**, and Yunnan **(D)**.

To investigate the presence of Foc TR4 we sampled commercial “Cavendish” plantations in Laos, Myanmar, Vietnam, and Yunnan during March and May 2016 (Table 1, Figure 1). Samples from Guangxi and Guangdong were collected during 2011–2014. Banana plants affected by Fusarium wilt which showed yellowing older leaves or a skirt of dead leaves around the pseudostem were internally sampled. Discolored vascular strands were collected from five plants at each location. Samples were wrapped in paper bags and maintained in a cool box until arrival in the laboratory.

**Table 1:** *Fusarium oxysporum* f.sp. *cubense* sampling code from Laos, Myanmar, Vietnam, and the Chinese provinces Yunnan, Guangxi.

**Sampling date**	**Site**	**Code**	**Variety**	**Location**	**Altitude (m)**
2016-05-11	Laos	La-1	Brazilian	21°25′32″N	580
				101°11′2″E	
2016-05-11	Laos	La-2	Brazilian	21°25′33″N	600
				101°11′2″E	
2016-05-11	Laos	La-3	Brazilian	21°25′33″N	590
				101°11′2″E	
2016-05-11	Laos	La-4	Brazilian	21°25′33″N	590
				101°11′3″E	
2016-05-11	Laos	La-5	Brazilian	21°25′34″N	590
				101°11′2″E	
2016-05-10	Myanmar	My-1	Brazilian	21°24′4″N	500
				100°23′4″E	
2016-05-10	Myanmar	My-2	Brazilian	21°24′3″N	490
				100°23′6″E	
2016-05-10	Myanmar	My-3	Brazilian	21°24′3″N	490
				100°23′6″E	
2016-05-10	Myanmar	My-4	Brazilian	21°24′ 5″N	510
				100°23′4″E	
2016-05-10	Myanmar	My-5	Brazilian	21°24′6″N	490
				100°23′4″E	
2016-03-17	Vietnam	VN-1	Guijiao No 6	22°30′42″N	102
				104°2′31″E	
2016-03-17	Vietnam	VN-2	Guijiao No 6	22°30′41″N	104
				104°2′31″E	
2016-03-17	Vietnam	VN-3	Guijiao No 6	22°30′39″N	108
				104°2′32″E	
2016-03-17	Vietnam	VN-4	Guijiao No 6	22°30′39″N	90
				104°2′32″E	
2016-03-23	Yunnan	YN-1	Nantianhuang	21°51′52″N	540
				100°56′17″E	
2016-03-23	Yunnan	YN-2	Nantianhuang	21°51′43″N	530
				100°56′13″E	
2016-03-23	Yunnan	YN-3	Nantianhuang	21°51′43″N	530
				100°56′13″E	
2016-03-23	Yunnan	YN-4	Nantianhuang	21°51′51″N	540
				100°56′23″E	
2016-03-23	Yunnan	YN-5	Nantianhuang	21°51′51″N	540
				100°56′23″E	
2012-02-16	Mengpeng, Yunnan	No. 3	Guijiao No 6	2l°30′32″N	550
				101°20′21″E	
2011-01-19	Puweng, Yunnan	No. 5	Brazilian	22°33′38″N	772
				101°23′37″E	
2013-11-24	Mengding, Yunnan	No. 6	Brazilian	23°28′34″N	450
				99°01′26″E	
2014-11-09	Wuming, Guangxi	N0. 16	Guangfen No. 1	23°16′37″N	150
				108°05′3″E	
2015-08-07	Pubei, Guangxi	No. 33	Xigong	22°13′36″N	56
				109°19′37″E	
2015-08-07	Lingshan, Guangxi	No. 34	Xigong	22°09′59″N	112
				109°13′40″E	

### Strain isolation and characterization

The collected samples were processed for Foc isolation and characterization as described earlier (Dita et al., 2010; García-Bastidas et al., 2014). Half of the dried vascular strands were placed on Komada medium (Leslie and Summerell, 2006) and the remaining part was used for DNA extraction to verify the presence of Foc TR4 (García-Bastidas et al., 2014; Ordonez et al., 2015a). Once purified single spore cultures were obtained, total DNA was isolated with the Wizard® Magnetic DNA Purification System for Food kit (Promega, Madison, USA)—following the manufacturer's instructions—for multiplex PCR analyses using diagnostic primers for Foc TR4 as well as for elongation factor-1α internal controls (Dita et al., 2010). Amplicons were visualized on agarose gels (1.2%, Roche, Mannheim, Germany) using an UV illuminator (Herolab, Wiesloch, Germany). Subsequently, one positive Foc TR4 strain for each country was phenotyped under greenhouse conditions (Unifarm, Wageningen, The Netherlands) following earlier reported protocols (García-Bastidas et al., 2014; Ordonez et al., 2015a). For each strain we used six highly susceptible “Grand Naine” plants (biological replicates) that were placed randomly in the greenhouse, along with the appropriate controls (negative: water and Foc Race 1 from Cruz das Almas, Brazil, positive: Foc TR4 reference isolate II5/VCG01213). The inoculated plants and the controls were monitored weekly and final external and internal scoring was conducted seven weeks after inoculation by a team of three experimentators according to previously reported protocols (García-Bastidas et al., 2014; Ordonez et al., 2015a). Corm tissue of each plant was collected and plated on Komada medium for fungal isolation and subsequent PCR confirmation of Foc TR4 as causal agent.

### Sequence analyses of Foc TR4 strains

To determine the identity of the strains and their relationship with other strains, one Foc TR4 strain from each country was arbitrarily selected for whole-genome sequencing at the Beijing Genome Institute (Hong Kong, China), using Illumina technology, yielding ~20 million cleaned reads (150 nt). To establish the phylogenetic relationship between the publically available *F. oxysporum* f.sp. *lycopersici* isolate Fol 4287 (Ma et al., 2010) and a range of Foc isolates (Table 2) we utilized the reference sequence alignment-based phylogeny builder (REALPHY; v. 1.11) (Bertels et al., 2014). As previously described (Woudenberg et al., 2015) for *Alternaria* genomes, Illumina generated short reads and sequence fragments (100 nt) derived from the previously assembled genomes (Fol4286 and Foc TR4 II5) were mapped against the Foc TR4 II5 reference genome using Bowtie2, followed by the extraction of high quality (default settings) polymorphic and non-polymorphic sites conserved in all analyzed isolates. The final pseudo-molecule was used to infer a maximum-likelihood phylogeny using PhyML with the generalized time reversible (GTR) nucleotide substitution model, and the robustness of the phylogeny was assessed using 500 bootstrap replicates.

**Table 2:** *Fusarium oxysporum* f.sp. *cubense* strains used for phylogenetic analysis.

***Fusarium oxysporum* f.sp. *cubense* isolate**	**Pathogenicity code**	**Origin**	**Source**
II-5	TR4	Indonesia	Dita et al., 2010
NRRL36102	Race 1	Brazil	Dita et al., 2010
B2	TR4	China	Guo et al., 2014
Pak1.1A	TR4	Pakistan	Ordonez et al., 2015a
Phi2.6C	TR4	Philippines	Ordonez et al., 2015a
Leb1.2C	TR4	Lebanon	Ordonez et al., 2015a
JV11	TR4	Jordan	García-Bastidas et al., 2014
My-1	TR4	Myanmar	This work
La-2	TR4	Laos	This work
VN-2	TR4	Vietnam	This work

Single-nucleotide polymorphisms (SNPs) were identified using GATK v3.3.0 (DePristo et al., 2011) by mapping short reads against the Foc TR4 II5 reference using BWA-mem, and duplicate reads were marked using Picard tools. Genomic variants were identified using GATK HaploTypeCaller, and a joint variant call set was generated using GATK GenotypeGVCFs. Subsequently, SNP variants were selected and filtered to retain high quality SNPs. These were used to determine the relationship between Foc TR4 isolates using a principle component analyses (PCA; R, adegenet package) and hierarchical clustering (UPGMA; R).

## Results

### Observation and sampling of fusarium wilt in the greater mekong subregion

**Figure 2:**
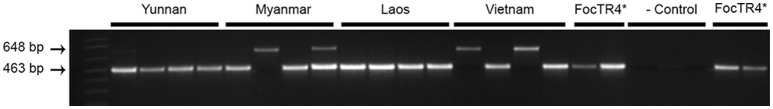
Identification of *Fusarium oxysporum* f.sp. *cubense* from samples derived from Yunnan, Myanmar, Laos, and Vietnam as Tropical Race 4 (Foc TR4) by PCR. Specific DNA bands for Foc TR4 (463 bp) and elongation factor-1α (648 bp) are indicated on the left. TR4-II5 was taken as positive control “Foc TR4*”; The Foc Race 1 strain was used as negative control (- control) (Dita et al., 2010).

In Laos and Myanmar, the predominant banana variety encountered in the plantations was the “Cavendish” variety “Brazilian,” while in the northern part of Vietnam “Cavendish” selection “Guijiao No. 6” was grown. Samples from Yunnan were collected in the Honghe and Xishuangbana prefectures in 2016 from the “Cavendish” varieties “Nantianhuang,” “Brazilian” or “Guijiao No. 6.” Fusarium wilt was observed in all plantations (Figure 1). In total 19 samples were collected; five samples from variety “Brazilian” in Laos and Myanmar, four samples from variety “Guijiao No 6” in Vietnam and five samples from variety “Nantianhuang” in Yunnan (Table 1, Figure 1). Analyses of the samples resulted in 16 isolates of which 13 were identified as Foc TR4 by diagnostic (463 bp) PCR reactions. The negative samples were positive for elongation factor-1α PCR (648 bp) and hence the DNA was present and of adequate quality. Positive controls yielded the diagnostic 463 bp PCR product and the negative controls did not show any DNA amplification (Figure 2).

### Phenotyping of Foc TR4 isolates

**Figure 3:**
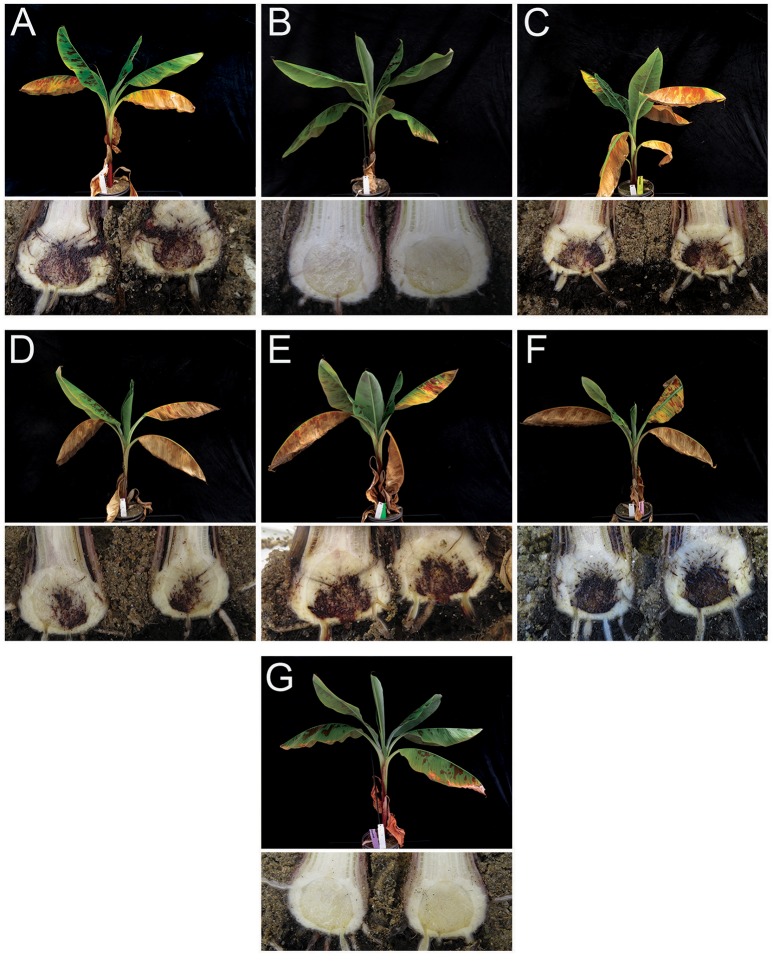
External foliar (top) and internal rhizome (bottom) symptoms of *Fusarium oxysporum* f.sp. *cubense* (Foc) infection in “Grand Naine” banana plants 7 weeks after inoculation using Foc isolates from Vietnam, Yunnan, Myanmar, and Laos. **(A)** Foc TR4 (reference Foc TR4, isolate II5 from Indonesia/VCG01213); **(B)** Foc Race 1 (from Cruz das Almas, Brazil); **(C)** Foc isolate from Xishuangbanna, Yunnan, China; **(D)** Foc isolate from Myanmar; **(E)** Foc isolate from Laos; **(F)** Foc isolate from Vietnam and; **(G)** Mock (water control).

We selected one Foc TR4 isolate from Vietnam, Yunnan, Myanmar, and Laos for confirmatory phenotyping assays. Except the control plants inoculated with Foc Race 1 or untreated controls (water), all inoculated “Grand Naine” plants showed typical external symptoms of Fusarium wilt starting from the fourth week after inoculation. The disease progressed steadily during incubation until plants were externally and internally scored for disease severity at seven weeks after inoculation. At that stage, all plants inoculated with Foc TR4 diagnosed strains were completely decayed. The Foc Race 1 inoculated plants, however, were healthy and unaffected by Foc, similar as the water controls (Figure 3).

**Figure 4:**
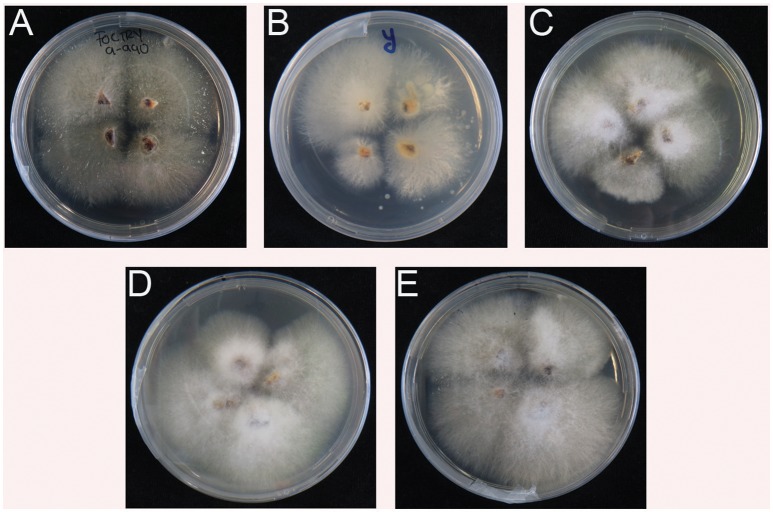
Re-isolation of *Fusarium oxysporum* f.sp. *cubense* TR4 from inoculated plants (see Figure 3). **(A–E)** from Indonesia, Yunnan, Myanmar, Laos, and Vietnam, respectively. No positive isolates for Foc TR4 were recovered from water and Foc Race 1 controls.

All inoculated plants and controls were sampled for another round of verification. In contrast to the controls (water and Race 1), all rhizomes from diseased plants enabled re-isolation of Foc. The resulting cultures showed typical Foc morphologies on Komada media (Figure 4) and subsequent Foc TR4 diagnostic PCR tests were positive for all reisolated strains (not shown).

### Sequence analysis of Foc TR4 strains

**Figure 5:**
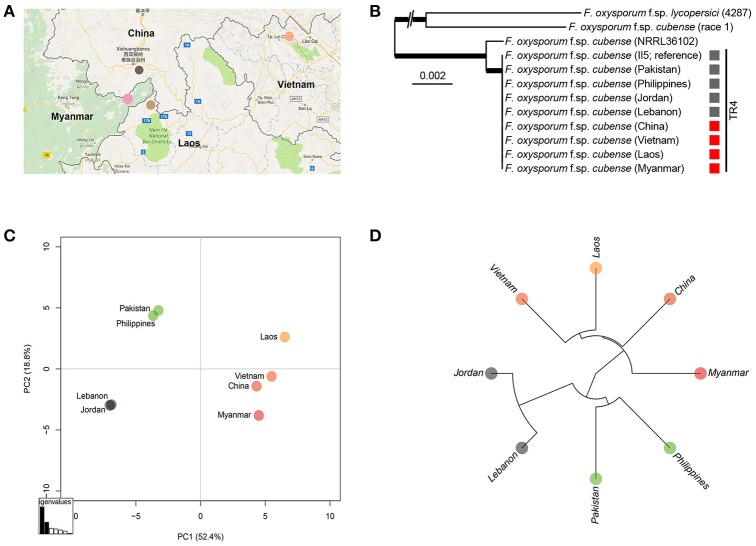
Sequence analysis of *Fusarium oxysporum* f.sp. *cubense* (Foc) isolates from Yunnan, Myanmar, Laos, and Vietnam and alignments with other Foc isolates from Jordan, Lebanon, Pakistan, and Philippines. **(A)** Colored dots represent sampling areas in China, Laos, Myanmar, and Vietnam; **(B)** Isolates from different countries; **(C)** Principal component analysis plot based on 251 high quality SNPs and; **(D)** UPGMA tree of Foc TR4 isolates.

We used whole-genome sequencing of each representative Foc TR4 strain for each GMS country and comparisons with the previously sequenced Foc TR4 strains from recently reported incursions were performed to study their genetic relatedness. The maximum-likelihood phylogeny of the genome sequences clearly confirmed that these strains belong to the Foc TR4 genetic lineage (Ordoñez et al., 2015b). Subsequent comparative analyses among the GMS Foc TR4 strains and those from recent incursions in Jordan, Lebanon, and Pakistan as well as a Philippine Foc TR4 strain revealed a total of 251 single nucleotide polymorphisms (SNPs) that were distributed across the genome (Supplemental Table 1, Supplemental Figure 1). Subsequent principal component analyses (PCA; Figure 5) and hierarchical clustering revealed three geographically distinct groups of Foc TR4 isolates, despite the overall limited amount of SNPs indicative of the clonality of Foc TR4 strains. One group represents the GMS Foc TR4 strains including the strain from Yunnan, China. A second group links the recent Pakistan incursion (Ordonez et al., 2015a) with the Philippine Foc TR4 strain. The third group shows a strong similarity between the recent incursion of Foc TR4 in Lebanon and Jordan (García-Bastidas et al., 2014; Ordonez et al., 2015a). Some of the analyzed SNPs were indicative of inconsistencies based on the FocII5 reference genome. Therefore, we further filtered the SNPs more stringently, yielding a subset of 161 SNPs (Supplemental Table 1). Overall, the resulting PCA and the hierarchical cluster (Supplemental Figures 2, 3) were nearly indistinguishable from the initial plots, thereby supporting the occurrence of three geographically distinct groups.

## Discussion

This study provides the earliest collected records of Foc TR4 in Vietnam and Laos and its first report in Myanmar. Recently, Mostert et al. (2017) denied the presence of Foc TR4 in Vietnam, Cambodia, and Thailand based on samples that were collected a decade ago, but (Chittarath et al., 2017; Hung et al., 2017) confirmed it in Vietnam and Laos. Our genome analyses revealed a set of SNPs that we used to further analyze the genetic diversity of Foc TR4 strains. Isolates from Vietnam, Laos, and Myanmar are genetically closely related and resemble the Foc TR4 strain from Yunnan. Furthermore, we demonstrate genetic association between the Foc TR4 strains from Pakistan and the Philippines as well as between the strains from Lebanon and Jordan. Although Foc TR4 is an asexually reproducing fungus which therefore shows a very strong linkage disequilibrium, clonal evolution does occur as evidenced by genetic clustering which enabled our biogeographical analysis (Tibayrenc and Ayala, 2012).

Recently, we demonstrated that the globally disseminating Foc TR4 strain represents essentially a single clone (Ordoñez et al., 2015b). Hence, it was difficult to unveil the origin of new incursions. However, we identified 251 high value SNPs that also after filtering elucidate basic associations between the here identified Foc TR4 strains. Such analyses were recently also used to reveal the dissemination of the quarantine pathogen *Xylella fastidiosa* in olive trees (Loconsole et al., 2014). Here, such a phylogeography approach provides initial evidence that Foc TR4 in Laos, Vietnam, and Myanmar was likely introduced from China. This supports the circumstantial evidence of ongoing Foc TR4 epidemics on “Cavendish” plantations in these countries and adjacent Chinese provinces, which were developed by Chinese banana entrepreneurs. The SNP analyses also revealed that the Foc TR4 strains from the Philippines and from Pakistan are closely related. Since Foc TR4 was diagnosed in the Philippines in 2005 (Molina et al., 2009) and is currently omnipresent in Mindanao, the recent incursion in Pakistan (Ordonez et al., 2015a) seems to originate from the Philippines. Similarly, the phylogeography data set indicates that the Foc TR4 incursions in Lebanon and Jordan are associated (García-Bastidas et al., 2014).

The introduction of large scale “Cavendish” monocultures in the GMS resulted in displacement of local peoples, disputes on landownership and also resulted in a rapid decrease in forest area, which challenges ecological stability (Rerkasem et al., 2009; Yoshida et al., 2010; Friis and Nielsen, 2016). We demonstrate that it also facilitated yet another expansion of Foc TR4. The dissemination of Foc TR4 in China upon the initial introduction from Taiwan is not well documented. Evidently, low awareness among banana growers and industry stakeholders has resulted in an almost unlimited movement of banana suckers, contaminated nursery soils, and farming equipment as well as the use of contaminated surface irrigation water. Our phylogeography approach indicates that these practices and the mobility of banana stakeholders may have contributed to the expansion of Foc TR4 (Drenth and Guest, 2016).

Similar to the previous Panama disease epidemic in “Gros Michel” caused by Foc Race 1 the lag phase of the current epidemic is substantial as the first occurrence of Foc TR4 was observed 50 years ago in Taiwan (Su et al., 1977, 1986; Hwang and Ko, 2004). However, the track-record of Fusarium wilt epidemics in banana is unparalleled in botanical epidemiology (Ploetz, 2015), and hence we should not underestimate the impact of the current Foc TR4 pandemic on food and fruit production. Despite numerous efforts to alert and mobilize the banana sector for enhanced quarantine practices, we observe a continuous dissemination of Foc TR4 (García-Bastidas et al., 2014; Ordonez et al., 2015a; Ploetz et al., 2015; Promusa, 2016; Chittarath et al., 2017; Hung et al., 2017). High resolution phylogeography may increase overall awareness and responsibility among banana stakeholders to prevent the further dissemination of Foc TR4.

## Author contributions

S-JZ: Conceived the experiments, collected the samples, analyzed the data, wrote the paper; FG-B: Analyzed the samples, wrote the paper; XL, LZ, TB, SX, KY, HL, YY, and LY: Collected the samples, analyzed the data; GF: Collected the samples, analyzed the data, wrote the paper; HN, BD, and AK: Ensured visits to banana farms, collected samples, organized permits; AD: Provided training, analyzed the data, wrote the paper; MS and HM: Analyzed the data, wrote the paper; GK: Conceived the experiments, organized the sampling, analyzed the data, wrote the paper.

### Conflict of interest statement

The authors declare that the research was conducted in the absence of any commercial or financial relationships that could be construed as a potential conflict of interest.

## Supplementary material

Supplemental Table 1 Distribution of 251 high quality SNPs over the genomes of the GMS Foc TR4 strains.

Supplemental Figure 1 Graphical representation of the distribution of 251 high quality SNPs over the genomes of the GMS Foc TR4 strains.

Supplemental Figure 2 Principal component analysis plot based on 161 filtered high quality SNPs of Foc TR4 isolates from Yunnan, Myanmar, Laos, Vietnam, Jordan, Lebanon, Pakistan, and Philippines.

Supplemental Figure 3 UPGMA tree based on 161 high quality SNPs over the genomes of the GMS Foc TR4 strains.
