# Supplementary material for: New Geographical Insights of the Latest Expansion of Fusarium oxysporum f.sp. cubense Tropical Race 4 Into the Greater Mekong Subregion
Source: Front Plant Sci. 2018 Apr 9;9:457. doi: 10.3389/fpls.2018.00457 (PMC5900031; doi:10.3389/fpls.2018.00457)
Supplement: Supplemental Table 1 — Distribution of 251 high quality SNPs over the genomes of the GMS Foc TR4 strains. [file Table1.PDF]

Supplemental Table 1. Distribution of 251 high quality SNPs distributed over the genomes of the GMS Foc TR4 strains.

Contigs	Position	Ref	Alt	Location	Retained after filtering	China	Jordan	Laos	Lebanon	Myanmar	Pakistan	Philippines	Vietnam
Supercontig_1.1	654887	G	C	intergenic	Y	1	0	1	0	1	0	0	1
Supercontig_1.1	1458700	A	G	intergenic	Y	1	0	1	0	1	0	0	1
Supercontig_1.1	1787865	C	T	FOIG_00628T4,FOIG_00628T1,	Y	1	0	1	0	1	0	0	1
Supercontig_1.1	2943895	A	G	FOIG_01024T0	Y	1	0	1	0	1	0	0	1
Supercontig_1.1	3247785	C	T	FOIG_01141T3,FOIG_01141T2,	Y	1	0	1	0	1	0	0	1
Supercontig_1.2	27307	T	C	FOIG_01589T0	Y	1	0	1	0	1	0	0	1
Supercontig_1.2	36211	C	A	FOIG_01593T0	Y	1	0	1	0	1	0	0	1
Supercontig_1.2	430585	C	A	FOIG_01750T0	Y	1	0	1	0	1	0	0	1
Supercontig_1.2	1264665	T	G	FOIG_02067T1,FOIG_02067T2,	Y	1	0	1	0	1	0	0	1
Supercontig_1.2	1352731	C	T	intergenic	Y	1	0	1	0	1	0	0	1
Supercontig_1.2	2122409	G	T	FOIG_02347T0	Y	1	0	1	0	1	0	0	1
Supercontig_1.3	309536	T	C	FOIG_02508T0	Y	1	0	1	0	1	0	0	1
Supercontig_1.3	371628	A	G	FOIG_02538T0	Y	1	0	1	0	1	0	0	1
Supercontig_1.3	371629	G	T	FOIG_02538T0	Y	1	0	1	0	1	0	0	1
Supercontig_1.3	1235003	T	C	intergenic	Y	1	0	1	0	1	0	0	1
Supercontig_1.4	513357	A	T	intergenic	Y	0	1	0	1	0	0	0	0
Supercontig_1.4	1121692	A	G	FOIG_03542T0,FOIG_03543T0	Y	1	0	1	0	1	0	0	1
Supercontig_1.4	1875736	C	A	intergenic	Y	1	0	1	0	1	0	0	1
Supercontig_1.4	1884466	T	C	intergenic	Y	1	0	1	0	1	0	0	1
Supercontig_1.4	1978399	A	C	FOIG_03881T0	Y	1	0	1	0	1	0	0	1
Supercontig_1.5	615490	G	A	FOIG_04148T2,FOIG_04148T0,	Y	1	0	1	0	1	0	0	1
Supercontig_1.5	758313	C	T	FOIG_04200T0	Y	1	0	1	0	1	0	0	1
Supercontig_1.5	850886	C	T	FOIG_04227T1,FOIG_04227T0	Y	1	0	1	0	1	0	0	1
Supercontig_1.5	920213	C	T	FOIG_04254T0,FOIG_04254T1	Y	1	0	1	0	1	0	0	1
Supercontig_1.6	780057	C	T	FOIG_04908T0	Y	1	0	1	0	1	0	0	1
Supercontig_1.6	1325607	A	G	FOIG_05103T3,FOIG_05103T0,	Y	0	1	0	1	0	0	0	0
Supercontig_1.6	1514485	G	A	FOIG_05161T0	Y	1	0	1	0	1	0	0	1
Supercontig_1.6	1692468	A	C	intergenic	Y	0	1	0	1	0	0	0	0
Supercontig_1.6	1702323	C	A	FOIG_05229T0,FOIG_05229T1	Y	1	0	1	0	1	0	0	1
Supercontig_1.7	1007173	C	G	intergenic	Y	0	1	0	1	0	0	0	0
Supercontig_1.8	94448	G	A	FOIG_05832T1,FOIG_05832T0	Y	1	0	1	0	1	0	0	1
Supercontig_1.8	241008	T	C	FOIG_05880T0	Y	1	0	1	0	1	0	0	1
Supercontig_1.8	1167657	T	C	intergenic	Y	1	0	1	0	1	0	0	1
Supercontig_1.8	1289418	T	C	FOIG_06248T1,FOIG_06248T0	Y	1	0	1	0	1	0	0	1
Supercontig_1.8	1337175	C	A	FOIG_06270T0	Y	1	0	1	0	1	0	0	1
Supercontig_1.9	1383195	C	A	FOIG_06887T0	Y	1	0	1	0	1	0	0	1
Supercontig_1.10	1090138	G	A	FOIG_07284T0	Y	1	0	1	0	1	0	0	1
Supercontig_1.11	742653	A	G	FOIG_07713T0,FOIG_07714T0	Y	1	0	1	0	1	0	0	1
Supercontig_1.13	2588	A	G	FOIG_08364T0	Y	1	0	1	0	1	0	0	1
Supercontig_1.13	1010783	G	A	FOIG_08801T0	Y	0	1	0	1	0	0	0	0
Supercontig_1.14	537513	A	G	FOIG_09012T0	Y	1	0	1	0	1	0	0	1
Supercontig_1.15	347201	C	A	FOIG_09319T0	Y	1	0	1	0	1	0	0	1
Supercontig_1.15	454571	C	T	FOIG_09372T0	Y	1	0	1	0	1	0	0	1
Supercontig_1.16	515283	G	T	FOIG_09770T0	Y	0	1	0	1	0	0	0	0
Supercontig_1.16	782381	A	G	intergenic	Y	1	0	1	0	1	0	0	1
Supercontig_1.17	331553	C	A	FOIG_10058T0	Y	1	0	1	0	1	0	0	1
Supercontig_1.18	12788	A	G	intergenic	Y	0	1	0	1	0	0	0	0
Supercontig_1.18	36789	G	A	intergenic	Y	1	0	1	0	1	0	0	1
Supercontig_1.18	362386	A	G	FOIG_10438T0	Y	1	0	1	0	1	0	0	1
Supercontig_1.20	555994	A	G	FOIG_11109T0	Y	1	0	1	0	1	0	0	1
Supercontig_1.21	143464	C	A	intergenic	Y	0	1	0	1	0	0	0	0
Supercontig_1.21	189518	A	G	intergenic	Y	1	0	1	0	1	0	0	1
Supercontig_1.22	186101	G	C	FOIG_11523T0	Y	1	0	1	0	1	0	0	1
Supercontig_1.23	465787	A	T	intergenic	Y	1	1	1	1	1	1	1	0
Supercontig_1.24	443510	T	A	FOIG_12076T0	Y	1	0	1	0	1	0	0	1
Supercontig_1.27	361683	A	G	FOIG_12607T0	Y	1	0	1	0	1	0	0	1
Supercontig_1.28	343198	C	T	intergenic	Y	1	0	1	0	1	0	0	1
Supercontig_1.30	321518	T	C	intergenic	Y	1	0	1	0	1	0	0	1
Supercontig_1.32	6817	C	A	intergenic	Y	1	0	1	0	1	0	0	1
Supercontig_1.32	327846	A	G	intergenic	Y	1	0	1	0	1	0	0	1
Supercontig_1.32	371795	A	G	FOIG_13366T0	N	1	0	1	0	1	1	1	1
Supercontig_1.33	67285	C	T	FOIG_13391T0	Y	1	0	1	0	1	0	0	1
Supercontig_1.33	258829	A	G	intergenic	Y	1	0	1	0	1	0	0	1
Supercontig_1.34	86818	C	A	FOIG_13549T0	Y	1	0	1	0	1	0	0	1
Supercontig_1.34	299791	T	C	FOIG_13634T0	Y	1	0	1	0	1	0	0	1
Supercontig_1.36	216436	C	G	FOIG_13836T0	Y	1	0	1	NA	1	0	0	1
Supercontig_1.38	2243	C	T	FOIG_14008T0,FOIG_14008T2,	Y	1	0	1	0	1	0	0	1
Supercontig_1.38	45829	A	T	intergenic	Y	1	0	1	0	1	0	0	1
Supercontig_1.38	193989	G	C	FOIG_14105T0	Y	1	0	1	0	1	0	0	1
Supercontig_1.39	227081	A	T	intergenic	Y	1	0	1	0	1	0	0	1
Supercontig_1.39	258411	G	A	FOIG_14277T0	Y	1	0	1	0	1	0	0	1
Supercontig_1.40	232563	C	T	FOIG_14377T0	Y	1	0	1	0	1	0	0	1
Supercontig_1.40	250319	A	G	intergenic	N	1	0	1	1	1	1	1	1
Supercontig_1.40	262562	C	T	intergenic	Y	1	0	1	0	1	0	0	1
Supercontig_1.43	238722	C	T	FOIG_14722T0,FOIG_14722T1	Y	1	0	1	0	1	0	0	1
Supercontig_1.44	237782	A	C	intergenic	Y	1	0	1	0	1	0	0	1
Supercontig_1.46	35988	G	T	FOIG_14954T0	Y	1	0	1	0	1	0	0	1
Supercontig_1.65	92080	G	A	FOIG_16094T0	Y	1	0	1	0	1	0	0	1
Supercontig_1.66	59047	G	A	FOIG_16120T0	Y	1	0	1	0	1	0	0	1
Supercontig_1.70	23683	G	A	intergenic	N	0	1	0	1	0	NA	0	0
Supercontig_1.70	24246	A	G	intergenic	N	1	1	1	0	1	1	1	1
Supercontig_1.70	26083	C	T	intergenic	N	1	1	0	1	1	0	1	1
Supercontig_1.70	32048	A	G	intergenic	Y	1	0	1	0	1	0	0	1
Supercontig_1.76	928	T	A	intergenic	N	1	1	0	1	1	0	NA	1

Supercontig_1.76	1155	A	G	intergenic	N	1	1	0	1	1	0	1	1
Supercontig_1.76	1191	T	A	intergenic	N	1	1	0	1	1	0	0	1
Supercontig_1.76	1364	G	A	intergenic	Y	0	1	0	1	1	0	0	0
Supercontig_1.76	1424	T	C	FOIG_16310T0	Y	1	1	0	1	1	0	0	0
Supercontig_1.76	1430	A	G	FOIG_16310T0	Y	0	1	0	1	1	0	0	0
Supercontig_1.76	1493	G	T	FOIG_16310T0	Y	1	1	0	1	1	0	0	0
Supercontig_1.76	1557	T	C	FOIG_16310T0	N	1	1	0	1	1	0	1	1
Supercontig_1.76	1595	C	T	FOIG_16310T0	N	1	1	0	1	1	NA	1	1
Supercontig_1.76	1654	C	T	FOIG_16310T0	N	0	1	0	1	1	1	0	1
Supercontig_1.76	1656	G	A	FOIG_16310T0	N	0	1	0	1	1	1	0	1
Supercontig_1.76	1766	T	C	FOIG_16310T0	Y	0	1	0	1	0	0	0	1
Supercontig_1.76	2063	G	A	intergenic	Y	0	1	0	1	0	1	1	0
Supercontig_1.76	2133	C	T	intergenic	Y	1	1	0	1	0	0	0	0
Supercontig_1.76	2152	T	G	intergenic	Y	1	1	0	1	0	0	1	0
Supercontig_1.76	2326	G	T	intergenic	Y	0	1	0	1	0	0	0	0
Supercontig_1.76	2448	T	G	intergenic	N	1	1	0	1	1	1	0	NA
Supercontig_1.76	2483	A	G	intergenic	N	1	1	0	1	1	1	0	1
Supercontig_1.76	2838	T	A	intergenic	Y	NA	1	0	1	0	1	0	0
Supercontig_1.76	3208	T	C	intergenic	N	1	1	0	1	1	1	1	0
Supercontig_1.76	3364	T	C	intergenic	Y	0	1	0	1	1	0	0	0
Supercontig_1.76	3956	T	C	intergenic	Y	NA	1	0	1	0	0	0	0
Supercontig_1.76	3965	G	T	intergenic	Y	NA	1	0	1	0	0	0	0
Supercontig_1.76	4176	G	A	intergenic	Y	0	1	0	1	1	1	0	0
Supercontig_1.76	4337	G	T	intergenic	N	1	1	0	1	1	0	1	1
Supercontig_1.76	4543	A	C	FOIG_16312T0	Y	0	1	0	1	0	0	0	NA
Supercontig_1.76	4630	A	G	FOIG_16312T0	N	1	1	0	1	0	1	0	1
Supercontig_1.76	4681	T	C	FOIG_16312T0	Y	1	1	0	1	0	1	0	0
Supercontig_1.76	4732	A	G	FOIG_16312T0	Y	0	1	0	1	0	1	0	0
Supercontig_1.76	4778	T	C	FOIG_16312T0	Y	0	1	0	1	0	0	1	NA
Supercontig_1.76	5167	G	A	intergenic	N	0	1	0	1	1	0	1	1
Supercontig_1.76	5417	C	A	intergenic	Y	0	1	0	1	0	0	1	1
Supercontig_1.76	5857	G	A	intergenic	N	1	1	0	1	0	1	1	0
Supercontig_1.76	5916	T	A	intergenic	Y	1	1	0	1	0	1	0	0
Supercontig_1.76	6054	C	T	intergenic	N	1	1	0	1	1	0	0	1
Supercontig_1.76	6442	T	C	FOIG_16313T0	Y	0	1	0	1	0	0	0	1
Supercontig_1.76	6481	T	G	FOIG_16313T0	Y	1	1	0	1	0	0	0	1
Supercontig_1.76	6568	C	T	FOIG_16313T0	N	1	1	0	1	1	1	0	0
Supercontig_1.76	6602	T	G	FOIG_16313T0	N	1	1	0	1	1	1	0	0
Supercontig_1.76	6658	C	T	FOIG_16313T0	Y	0	1	0	1	0	0	1	0
Supercontig_1.76	6742	T	C	FOIG_16313T0	Y	0	1	0	1	NA	0	1	0
Supercontig_1.76	6859	T	C	FOIG_16313T0	Y	1	1	0	1	1	0	0	0
Supercontig_1.76	6928	A	C	FOIG_16313T0	Y	NA	1	0	1	1	0	0	0
Supercontig_1.76	7774	C	T	FOIG_16313T0	Y	0	1	0	1	0	1	1	0
Supercontig_1.76	8448	C	T	intergenic	N	1	1	0	1	1	1	1	1
Supercontig_1.76	8842	A	G	FOIG_16314T0	Y	1	1	0	1	0	1	0	0
Supercontig_1.76	9131	T	A	FOIG_16314T0	N	1	1	0	1	1	0	1	0
Supercontig_1.76	9144	A	G	FOIG_16314T0	N	1	1	0	1	1	0	1	0
Supercontig_1.76	9199	A	G	FOIG_16314T0	N	1	1	0	1	1	0	1	0
Supercontig_1.76	9391	A	G	FOIG_16314T0	Y	1	1	0	1	0	0	0	0
Supercontig_1.76	9474	G	A	FOIG_16314T0	Y	0	1	0	1	0	0	1	0
Supercontig_1.76	10604	C	G	intergenic	N	0	1	0	1	0	1	1	1
Supercontig_1.76	11524	G	T	FOIG_16315T0	N	1	1	0	1	0	0	1	1
Supercontig_1.76	11566	C	T	FOIG_16315T0	N	1	1	0	1	0	0	1	1
Supercontig_1.76	11790	G	T	FOIG_16315T0	N	1	1	0	1	1	0	1	NA
Supercontig_1.76	11847	C	T	FOIG_16315T0	N	1	1	0	1	1	0	1	0
Supercontig_1.76	12165	C	T	FOIG_16315T0	Y	0	1	0	1	0	0	1	0
Supercontig_1.76	12296	C	T	FOIG_16315T0	Y	0	1	0	1	0	0	1	0
Supercontig_1.76	13808	T	C	intergenic	Y	1	1	0	1	1	0	0	0
Supercontig_1.76	14444	T	C	FOIG_16316T0	Y	0	1	0	1	1	0	0	0
Supercontig_1.76	14891	A	G	FOIG_16316T0	N	NA	1	0	1	1	0	1	0
Supercontig_1.76	15301	T	C	FOIG_16316T0	N	1	1	0	1	0	0	1	1
Supercontig_1.76	17607	A	G	FOIG_16317T0	Y	0	1	0	1	0	0	0	0
Supercontig_1.76	18313	T	C	FOIG_16317T0	Y	0	1	0	1	0	0	1	1
Supercontig_1.76	18398	G	A	FOIG_16317T0	N	1	1	0	1	1	0	1	1
Supercontig_1.76	18405	A	T	FOIG_16317T0	N	1	1	0	1	1	1	1	1
Supercontig_1.76	18561	A	G	FOIG_16317T0	N	1	1	0	1	1	1	1	1
Supercontig_1.76	18645	G	A	FOIG_16317T0	N	1	1	0	1	0	0	1	NA
Supercontig_1.76	18769	C	T	FOIG_16317T0	N	1	1	0	1	0	0	1	1
Supercontig_1.76	19470	G	A	intergenic	Y	0	1	0	1	0	0	0	0
Supercontig_1.76	20306	T	G	FOIG_16318T0	Y	1	1	0	1	0	0	0	NA
Supercontig_1.76	20603	T	G	FOIG_16318T0	N	1	1	0	1	1	0	0	1
Supercontig_1.76	20656	G	A	FOIG_16318T0	N	1	1	0	1	1	0	0	1
Supercontig_1.76	20663	T	C	FOIG_16318T0	N	1	1	0	1	1	0	0	1
Supercontig_1.76	20745	G	A	FOIG_16318T0	N	1	1	0	1	0	1	1	NA
Supercontig_1.76	22394	T	C	intergenic	Y	1	1	0	1	0	1	0	0
Supercontig_1.76	22424	A	G	intergenic	Y	0	1	0	1	1	1	0	0
Supercontig_1.76	22700	A	G	FOIG_16319T0	N	1	1	0	1	1	0	0	1
Supercontig_1.76	22708	C	T	FOIG_16319T0	N	1	1	0	1	1	0	0	1
Supercontig_1.76	22848	A	C	FOIG_16319T0	Y	0	1	0	1	0	1	0	1
Supercontig_1.76	22854	A	G	FOIG_16319T0	Y	0	1	0	1	0	1	0	1
Supercontig_1.76	23330	C	A	intergenic	N	0	1	0	1	1	1	0	1
Supercontig_1.76	25265	C	G	intergenic	Y	1	1	0	1	1	1	1	1
Supercontig_1.76	26728	T	C	FOIG_16320T0	Y	0	1	0	1	NA	0	0	0
Supercontig_1.76	27494	T	C	FOIG_16320T0	N	0	1	0	1	1	1	1	1
Supercontig_1.76	27596	T	G	FOIG_16320T0	Y	0	1	0	1	1	0	0	0
Supercontig_1.84	15215	G	C	FOIG_16465T0	Y	1	0	NA	0	1	0	0	1
Supercontig_1.84	27077	G	A	FOIG_16470T0	Y	1	0	NA	0	1	0	0	1
Supercontig_1.85	13391	A	G	intergenic	N	0	1	1	1	1	NA	0	1
Supercontig_1.85	13403	G	A	intergenic	N	0	1	1	1	1	0	0	1

Supercontig_1.85	24733	C	T	intergenic	N	0	1	1	1	1	0	0	0
Supercontig_1.85	24795	C	T	intergenic	N	1	1	1	1	1	0	1	0
Supercontig_1.85	24814	C	T	intergenic	N	1	1	1	1	1	1	0	0
Supercontig_1.85	24900	C	T	intergenic	N	1	1	0	1	NA	1	0	0
Supercontig_1.85	52471	A	G	intergenic	Y	1	0	1	0	1	0	0	1
Supercontig_1.86	2558	A	G	intergenic	N	1	1	1	1	1	0	1	1
Supercontig_1.86	19596	A	G	FOIG_16502T0	N	1	1	1	0	1	1	1	1
Supercontig_1.90	16313	A	T	intergenic	N	0	1	1	NA	1	1	1	0
Supercontig_1.91	14311	A	G	intergenic	Y	1	0	NA	0	1	0	0	1
Supercontig_1.91	19872	C	T	FOIG_16548T0	Y	1	0	NA	0	1	0	0	1
Supercontig_1.92	28236	C	T	intergenic	N	0	1	1	1	0	1	0	1
Supercontig_1.94	532	C	T	intergenic	Y	1	0	1	0	1	1	1	1
Supercontig_1.94	573	A	G	FOIG_16574T0	Y	1	0	1	0	1	1	1	1
Supercontig_1.94	12623	T	C	intergenic	Y	1	0	1	1	1	1	1	1
Supercontig_1.94	25913	T	G	FOIG_16582T0	Y	1	0	1	1	1	1	1	1
Supercontig_1.94	35440	C	T	intergenic	Y	1	0	1	0	1	1	1	1
Supercontig_1.94	37259	C	T	intergenic	Y	1	0	NA	1	1	1	1	1
Supercontig_1.94	37273	A	T	intergenic	Y	1	0	NA	1	1	1	1	1
Supercontig_1.99	26154	C	A	intergenic	Y	1	0	1	0	1	0	0	1
Supercontig_1.99	26251	T	C	intergenic	Y	1	0	1	0	1	0	0	1
Supercontig_1.99	26253	A	C	intergenic	Y	1	0	1	0	1	0	0	1
Supercontig_1.99	26389	T	C	intergenic	Y	NA	0	1	0	1	0	0	1
Supercontig_1.101	7813	C	A	intergenic	N	0	0	1	0	1	0	0	1
Supercontig_1.101	7820	A	G	intergenic	N	0	0	1	0	1	0	0	1
Supercontig_1.102	15860	A	G	intergenic	Y	1	0	1	0	1	0	0	1
Supercontig_1.102	19881	T	C	FOIG_16635T0	Y	1	0	1	0	1	0	0	1
Supercontig_1.107	20397	C	T	intergenic	N	0	1	1	1	1	0	1	1
Supercontig_1.112	3316	A	G	intergenic	N	1	1	0	1	0	1	1	0
Supercontig_1.112	9359	T	A	FOIG_16669T0	N	1	1	NA	1	0	1	1	1
Supercontig_1.117	10380	A	G	intergenic	N	1	1	1	1	0	1	1	1
Supercontig_1.122	11518	C	T	FOIG_16695T0,FOIG_16695T1	Y	1	0	1	0	1	0	0	1
Supercontig_1.128	5352	G	T	FOIG_16710T0	N	0	0	1	0	1	1	1	1
Supercontig_1.128	7462	A	G	intergenic	N	1	1	0	1	0	1	1	1
Supercontig_1.128	7467	G	A	intergenic	N	1	1	0	1	0	1	1	0
Supercontig_1.128	7707	T	A	intergenic	N	1	1	0	1	1	1	0	1
Supercontig_1.128	7757	A	G	intergenic	N	1	1	0	1	1	1	0	1
Supercontig_1.128	7826	A	G	intergenic	N	1	1	0	1	1	1	0	1
Supercontig_1.128	7954	G	T	FOIG_16711T0	N	1	1	0	1	0	1	0	0
Supercontig_1.128	8613	T	C	FOIG_16711T0	N	0	1	0	1	0	1	0	1
Supercontig_1.128	8985	C	T	FOIG_16711T0	N	0	1	0	1	1	1	1	0
Supercontig_1.128	8998	T	G	FOIG_16711T0	N	0	1	0	1	1	1	0	0
Supercontig_1.128	9053	G	A	FOIG_16711T0	N	0	1	0	1	1	1	0	0
Supercontig_1.128	9137	A	G	FOIG_16711T0	N	0	1	0	1	NA	1	0	1
Supercontig_1.128	9346	C	A	FOIG_16711T0	N	0	1	0	1	0	1	1	1
Supercontig_1.128	10408	T	G	intergenic	N	1	1	0	1	1	1	NA	1
Supercontig_1.128	10414	C	G	intergenic	N	1	1	0	1	1	1	0	1
Supercontig_1.128	10713	A	G	intergenic	Y	0	1	0	1	0	1	0	0
Supercontig_1.144	9283	G	A	intergenic	N	0	0	1	0	1	NA	0	1
Supercontig_1.190	1642	T	C	intergenic	N	0	1	1	1	1	0	0	1
Supercontig_1.190	1678	A	G	intergenic	N	1	1	1	1	1	1	0	1
Supercontig_1.190	1834	C	T	intergenic	N	1	1	1	1	1	1	0	1
Supercontig_1.190	1952	A	G	intergenic	N	1	1	1	1	1	1	0	1
Supercontig_1.190	2003	A	G	intergenic	N	NA	1	1	1	1	1	0	0
Supercontig_1.190	2049	A	C	intergenic	N	1	1	1	1	0	1	0	0
Supercontig_1.190	2204	C	T	intergenic	N	1	1	NA	1	1	1	0	0
Supercontig_1.190	2462	A	G	intergenic	N	1	1	1	1	1	1	0	1
Supercontig_1.219	2379	T	C	intergenic	N	0	0	1	0	1	0	0	1
Supercontig_1.219	2418	G	T	intergenic	N	0	0	1	0	1	0	0	1
Supercontig_1.219	2456	T	C	intergenic	N	0	0	1	0	1	0	0	1
Supercontig_1.219	2477	T	C	intergenic	N	0	0	1	0	1	0	0	1
Supercontig_1.264	262	G	A	intergenic	Y	0	1	0	1	1	0	1	0
Supercontig_1.264	309	T	G	intergenic	Y	0	1	0	1	1	0	0	0
Supercontig_1.264	543	G	A	intergenic	Y	0	1	0	1	1	0	0	0
Supercontig_1.264	614	G	C	intergenic	Y	0	1	0	1	1	0	0	0
Supercontig_1.264	777	C	T	intergenic	Y	0	1	0	1	1	0	0	0
Supercontig_1.264	953	A	G	intergenic	Y	0	1	0	1	1	0	1	0
Supercontig_1.264	1038	A	C	intergenic	Y	0	1	0	1	1	0	1	0
Supercontig_1.264	1060	C	T	intergenic	Y	0	1	0	1	1	0	1	0
Supercontig_1.264	1118	G	A	intergenic	Y	0	1	0	1	1	0	1	0
Supercontig_1.264	1157	A	G	intergenic	Y	0	1	0	1	1	0	0	0
Supercontig_1.264	1556	C	T	intergenic	Y	0	1	0	1	1	0	1	0
Supercontig_1.264	1585	G	A	intergenic	Y	0	1	0	1	1	0	0	0
Supercontig_1.264	1608	G	A	intergenic	Y	0	1	0	1	1	0	1	0
Supercontig_1.264	1661	A	G	intergenic	Y	0	1	0	1	1	0	1	0
Supercontig_1.264	1888	A	G	intergenic	Y	0	1	0	1	1	0	1	1
Supercontig_1.264	2010	G	T	intergenic	Y	0	1	0	1	1	0	1	0
Supercontig_1.354	776	G	A	FOIG_16942T0	Y	1	0	1	0	1	0	0	1
Supercontig_1.360	199	C	T	FOIG_16945T0	Y	1	1	1	1	0	1	1	0
