# Supplementary figures and images for: New Geographical Insights of the Latest Expansion of Fusarium oxysporum f.sp. cubense Tropical Race 4 Into the Greater Mekong Subregion
Source: Front Plant Sci. 2018 Apr 9;9:457. doi: 10.3389/fpls.2018.00457 (PMC5900031; doi:10.3389/fpls.2018.00457)

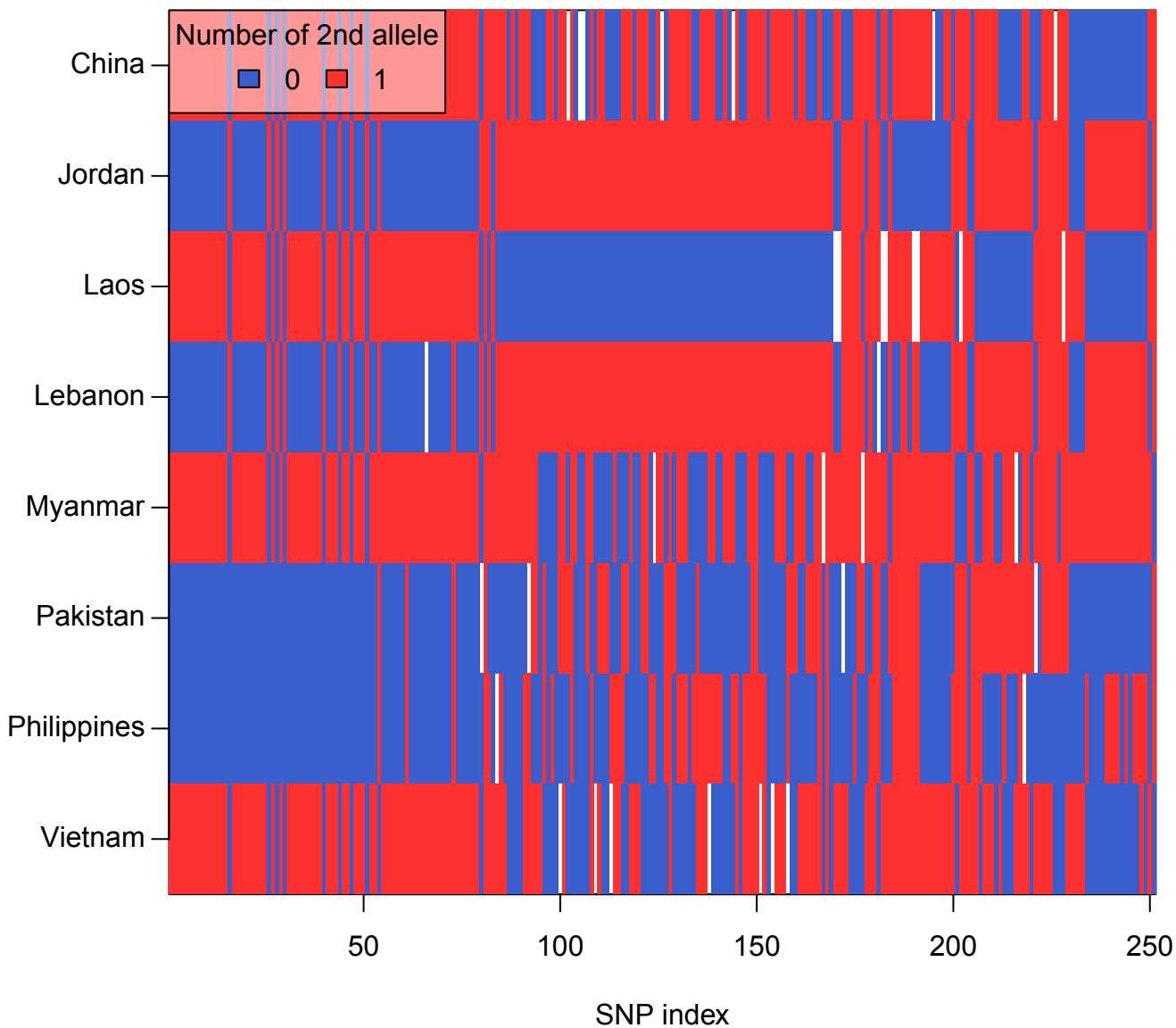

Supplement: Supplemental Figure 1 — Graphical representation of the distribution of 251 high quality SNPs over the genomes of the GMS Foc TR4 strains. [file Image1.PDF]

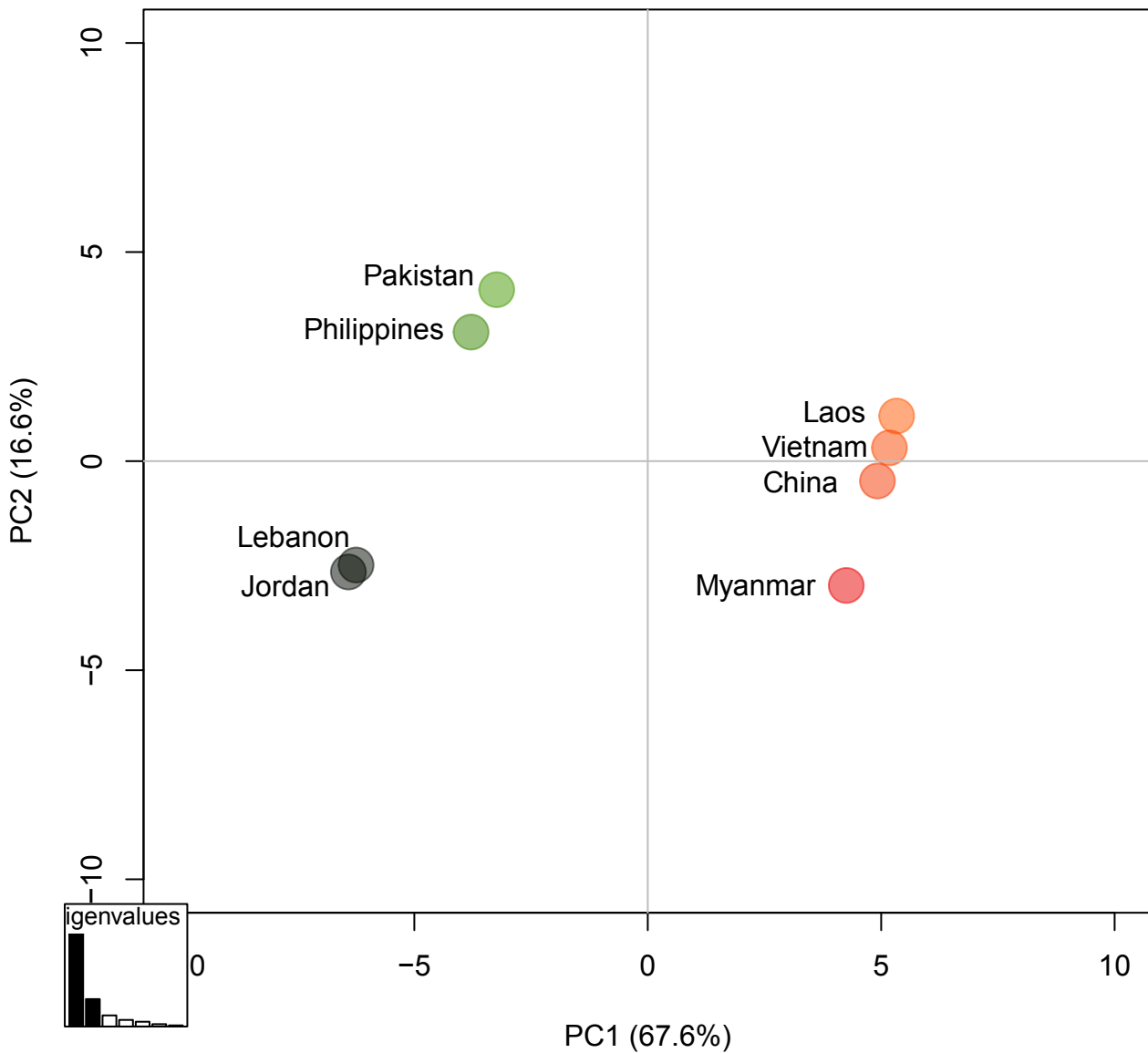

Supplement: Supplemental Figure 2 — Principal component analysis plot based on 161 filtered high quality SNPs of Foc TR4 isolates from Yunnan, Myanmar, Laos, Vietnam, Jordan, Lebanon, Pakistan, and Philippines. [file Image2.PDF]

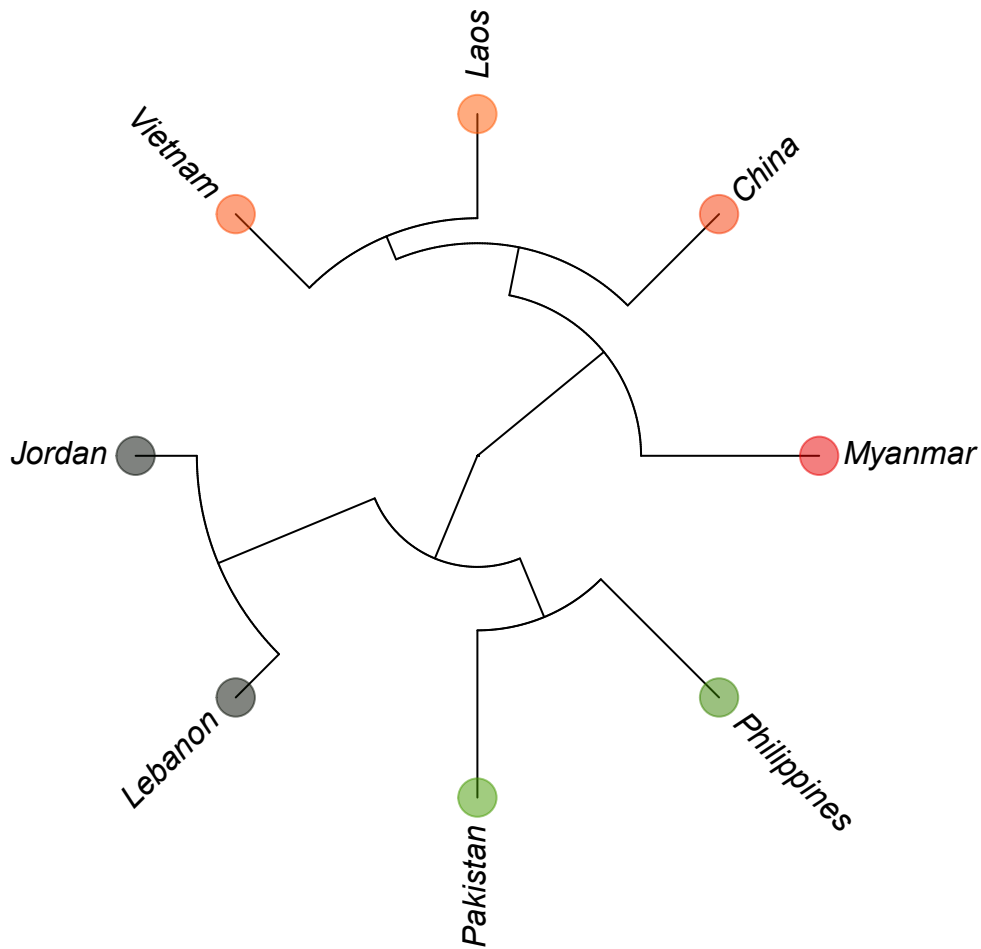

Supplement: Supplemental Figure 3 — UPGMA tree based on 161 high quality SNPs over the genomes of the GMS Foc TR4 strains. [file Image3.PDF]
